# Integrated bioinformatics analysis of the transcription factor-mediated gene regulatory networks in the formation of spermatogonial stem cells

**DOI:** 10.3389/fphys.2022.949486

**Published:** 2022-12-08

**Authors:** Kesong Shi, Baoluri Wang, Le Dou, Shu Wang, Xinrui Fu, Haiquan Yu

**Affiliations:** State Key Laboratory of Reproductive Regulation and Breeding of Grassland Livestock (RRBGL), Inner Mongolia University, Hohhot, China

**Keywords:** gene regulatory networks, transcription factor, spermatogonial stem cells, ATAC-seq, DNase-seq, WGCNA

## Abstract

**Background:** *In vitro* induction of spermatogonial stem cells (SSCs) from embryonic stem cells (ESCs) provides a promising tool for the treatment of male infertility. A variety of molecules are involved in this complex process, which needs to be further clarified. Undoubtedly, the increased knowledge of SSC formation will be beneficial to facilitate the currently complex induction process.

**Methods:** Based on ATAC-seq, DNase-seq, RNA-seq, and microarray data from GEO datasets, chromatin property data (ATAC-seq, DNase-seq) and gene expression data (RNA-seq, microarray data) were combined to search for SSC-specific transcription factors (TFs) and hub SSC-specific genes by using the WGCNA method. Then, we applied RNA-seq and microarray data screening for key SSC-specific TFs and constructed key SSC-specific TF-mediated gene regulatory networks (GRNs) using ChIP-seq data.

**Results:** First, after analysis of the ATAC-seq and DNase-seq data of mouse ESCs, primordial germ cells (PGCs), and SSCs, 33 SSC-specific TFs and 958 targeting genes were obtained. RNA-seq and WGCNA revealed that the key modules (turquoise and red) were the most significantly related to 958 SSC-specific genes, and a total of 10 hub SSC-specific genes were identified. Next, when compared with the cell-specific TFs in human ESCs, PGCs, and SSCs, we obtained five overlapping SSC-specific TF motifs, including the NF1 family TF motifs (NFIA, NFIB, NFIC, and NFIX), GRE, Fox:Ebox, PGR, and ARE. Among these, *Nfib* and *Nfix* exhibited abnormally high expression levels relative to mouse ESCs and PGCs. Moreover, *Nfib* and *Nfix* were upregulated in the testis sample with impaired spermatogenesis when compared with the normal group. Finally, the ChIP-seq data results showed that NFIB most likely targeted the hub SSC-specific genes of the turquoise module (*Rpl36al*, *Rps27*, *Rps21*, *Nedd8*, and *Sec61b*) and the red module (*Vcam1* and *Ccl2*).

**Conclusion:** Our findings preliminarily revealed cell-specific TFs and cell-specific TF-mediated GRNs in the process of SSC formation. The hub SSC-specific genes and the key SSC-specific TFs were identified and suggested complex network regulation, which may play key roles in optimizing the induction efficiency of the differentiation of ESCs into SSCs *in vitro*.

## Introduction

Infertility is a major health problem worldwide, and it is estimated that infertility affects approximately 10% of couples globally, with the male factor being a primary or contributing cause in approximately 50% of couples (Li et al., 2019; Ishikura et al., 2021). Male infertility is a multifactorial pathological condition in which genetic factors are highly complex, and azoospermia is the most common genetic factors that contributes to male infertility (Krausz and Riera-Escamilla, 2018). Spermatogonial stem cells (SSCs) are the ancestral cells of sperm and are the basis for spermatogenesis and fertility in men (Kossack et al., 2009). Therefore, SSCs are considered a promising alternative for the regeneration of impaired or damaged spermatogenesis, and SSCs transplantation is a promising technique for male infertility treatment (Abdelaal et al., 2021). Nevertheless, the number of SSCs is very scarce, and long-term culture and expansion of SSCs have not yet been available (Zhou et al., 2018). At present, both in mice and humans, several studies have found that ESCs have the ability to form putative primordial germ cells (PGCs), and these ESC-derived PGCs could further differentiate into SSCs (Nagamatsu and Hayashi, 2017; Li et al., 2019; Ishikura et al., 2021). However, these reports either involve a complex induction process with undefined induction factors or show a low induction efficiency, while the reconstitution of SSC formation *in vitro* remains a key challenge (Nagamatsu and Hayashi, 2017).

The dynamic reorganization of chromatin is accompanied by a genome-wide transcriptional change. In recent years, chromatin accessibility profiling has become an important tool for studying genetic and epigenetic regulation (Maezawa et al., 2018), allowing us to dissect the pangenomic regulatory landscape of cells and tissues in both time and space dimensions by detecting specific chromatin states and their corresponding TFs (Ma and Zhang, 2020). Moreover, chromatin accessibility profiling is expected to be a powerful tool for the identification of regulatory DNA elements that gene regulatory networks (GRNs), and changes in chromatin accessibility can be interpreted in the context of these dynamic regulatory networks (Nikolaev et al., 2007). The assay for transposase-accessible chromatin with high-throughput sequencing (ATAC-seq) and DNase I hypersensitive sites sequencing (DNase-seq) are a technology that maps the landscape of chromatin accessibility (Shashikant and Ettensohn, 2019). This method not only recognizes different cell types but also reveals cell-type-specific regulatory regions and detects the chromatin accessibility of related genes and putative TF-binding sites (Buenrostro et al., 2013; Shashikant and Ettensohn, 2019). Chromatin accessibility is closely correlated with the differential expression of genes, and it can potentially be a transcription factor regulator (Huang et al., 2020). Recently, ATAC-seq and ChIP-seq were combined to profile the change in chromatin accessibility in spermatogenesis. Namekawa et al. revealed the genome-wide, dynamic reorganization of open chromatin during spermatogenesis and detected possible regulatory elements for spermatogenesis-specific gene expression (Maezawa et al., 2018). They also found distinct chromatin environments of autosomes and sex chromosomes during spermatogenesis, suggesting that poised chromatin and the formation of bivalent domains underlie genome-wide epigenetic changes during late spermatogenesis (Sin et al., 2015).

Gene regulatory networks of the cells can also be revealed through chromatin profiling assays. Currently, GRNs have been used in many different areas, such as B-cell differentiation in the mammalian immune system (Singh et al., 2005), ankylosing spondylitis (Yu et al., 2020), plasma cell function (Trezise and Nutt, 2021) and immune cells associated with cancer (Han et al., 2017). Among them, through systematic analysis of the GRNs of immune cells, our group’s previous study found that the network size of the GRNs of tumour-infiltrating immune T cells was reduced when compared to the GRNs of their corresponding immune cells in blood (Han et al., 2017). It is already known that the transcription factors Plzf and Taf4b have been implicated in regulating SSC functions, and these molecules are part of a robust gene network controlling SSC fate decisions (Oatley and Brinster, 2008). However, the intrinsic GRNs that control SSC fate decisions and that disrupt these networks in clinical cases of human male infertility have yet to be determined (Oatley and Brinster, 2008).

**FIGURE 1:**
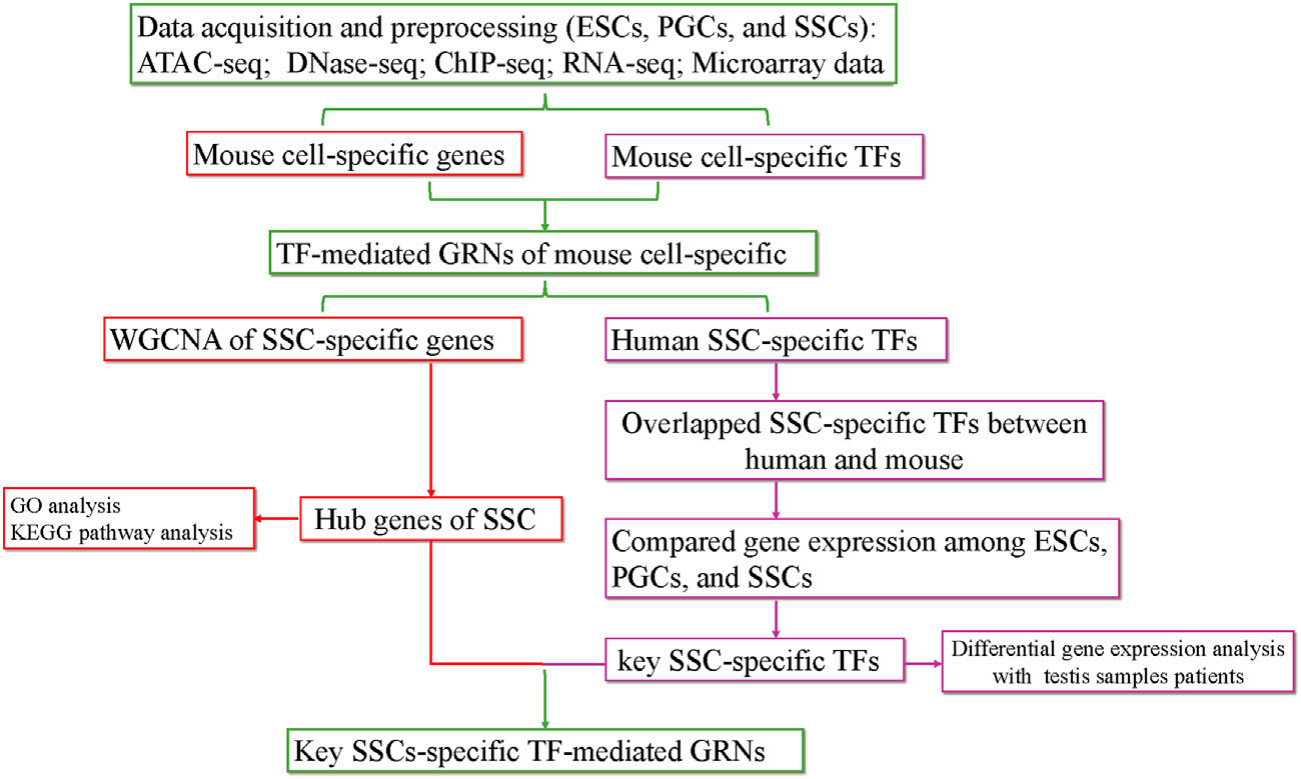
The flow chart of the analysis process. KEGG, Kyoto Encyclopedia of Genes and Genomes; GO, Gene Ontology.

The purpose of our study was to screen for factors that induce the differentiation of ESCs into SSCs *in vitro* by constructing TF-mediated GRNs during the formation of SSCs. First, we searched for SSC-specific TFs and hub SSC-specific genes based on chromatin property data (ATAC-seq, DNase-seq) and gene expression data (RNA-seq, microarray data). Then, we obtained overlapping SSC-specific TFs between humans and mice. Next, key SSC-specific TFs were obtained by comparing gene expression among ESCs, PGCs, and SSCs. Finally, we analysed the gene expression levels of key SSC-specific TFs in testis samples with impaired spermatogenesis compared with the normal group and constructed key SSC-specific TF-mediated GRNs using ChIP-seq data (Figure 1). Our study provides potential induction factors for further optimizing the induction efficiency of the differentiation of ESCs into SSCs *in vitro.*

## Materials and methods

### Data sources

The ATAC-seq, DNase-seq, RNA-seq, and microarray data were obtained from the GEO database (http://www.ncbi.nlm.nih.gov/geo). ChIP-seq data from the Cistrome (http://www.cisttrome.org/db). The details are shown in Supplementary Table S1.

### ATAC-seq, DNase-seq and regulatory network construction

To explore the accessibility of chromatin and obtain cell-specific TFs, raw sequence reads were initially processed by FastQC v0.11.9 for quality control, and quality filtering and adapter trimming were performed using Cutadapt v1.9. Then, reads were aligned to the reference genome (mm10 or hg38) with Bowtie2 (v2.4.4) (Langmead and Salzberg, 2012). Bam files from the resulting sam files by using Samtools v1.9 (Li et al., 2009). The peaks that replicated across each sample were merged into a single file using the bedtools software v2.30.0 (Quinlan and Hall, 2010). Peaks with an initial threshold *q*-value of 0.05 as the cut-off in experimental bam files were called by using MACS2 v2.2.7.1 (Zhang et al., 2008), and the peaks were identified with the parameters callpeak --shift -100 --extsize 200. DeepTools v2.0 (Ramírez et al., 2014) was used to enrich peaks in the transcriptional start site (TSS) region by using the computeMatrix and plotHeatmap functions. Annotation of peaks was performed using the ChIPSeeker package (Yu et al., 2015). Library to annotate the peaks to genomic features using a cut-off of 2 kb ± from TSS. Subsequently, we applied HOMER’s FindMotifGenome.pl tool v4.11 (Heinz et al., 2010) to obtain the list of TFs that putatively bind to the peaks. The TFs were filtered based on the *p-value*, and the final results were filtered based on the *p-value* ≤ 1 × 10^–4^. To discover the TFs that can bind to cell-specific genes, we used ChIP-seq data from Cistrome Data (score >1). The TFs and their regulated downstream genes were confirmed, and GRNs were constructed. GRNs and regulatory circuit analyses were conducted by Cytoscape v3.7.2 software. For the visualization of read count data, bam files, bigwig files, and genome browser images were made using the Integrative Genomics Viewer (IGV) tools (Thorvaldsdóttir et al., 2013). The UpSet diagram was generated using the R package UpSetR v1.4.0.

### RNA-seq and microarray data analysis

The raw reads were processed as given above. High-quality reads were then mapped to the mm10 reference genome using HISAT2 v2.2.1 (Kim et al., 2015). Aligned RNA-seq reads were quantified and annotated using featureCounts (Liao et al., 2014), and gene expression levels were calculated based on RNA-seq data as transcripts per kilobase million (TPM) values, and TPM values from RNA-seq samples were averaged. Microarray data form GSE145467 (http://www.ncbi.nlm.nih.gov/geo), which contained 20 samples (10 testis samples with normal spermatogenesis and 10 testis samples with impaired spermatogenesis). Differential gene expression analysis was performed using GEO2R (https://www.ncbi.nlm.nih.gov/geo/geo2r). Volcano plots were plotted using the ggplot2 package (http://ggplot2.org/). Heatmaps were created using the pheatmap package v1.0.12 (https://CRAN.R-project.org/package=pheatmap).

### WGCNA, hub gene selection and enrichment analysis

In this study, the coexpressed gene module and the hub module correlated with SSCs were analysed using the R package WGCNA (Langfelder and Horvath, 2008), and we selected 958 SSC-specific genes targeted by SSC-specific TFs for WGCNA network construction. A power of β = 9 and the adjacencies between all the filtered genes were transformed into the corresponding dissimilarity. The hub module and hub genes were visualized with the plug-in MCODE of Cytoscape v3.7.2 with a cut-off MCODE score of ≥ 4.5. Afterwards, the functional annotation and pathway enrichment for the genes in the hub module was conducted using DAVID (false-discovery rate, *FDR* < 0.05) (Huang da et al., 2009), and Kyoto Encyclopedia of Genes and Genomes (KEGG) pathway and Gene Ontology (GO) analyses were plotted using R package GOplot v1.0.2 (https://cran.r-project.org/web/packages//GOplot/).

## Results

### Mapping accessible chromatin in mouse ESCs, PGCs, and SSCs

**FIGURE 2:**
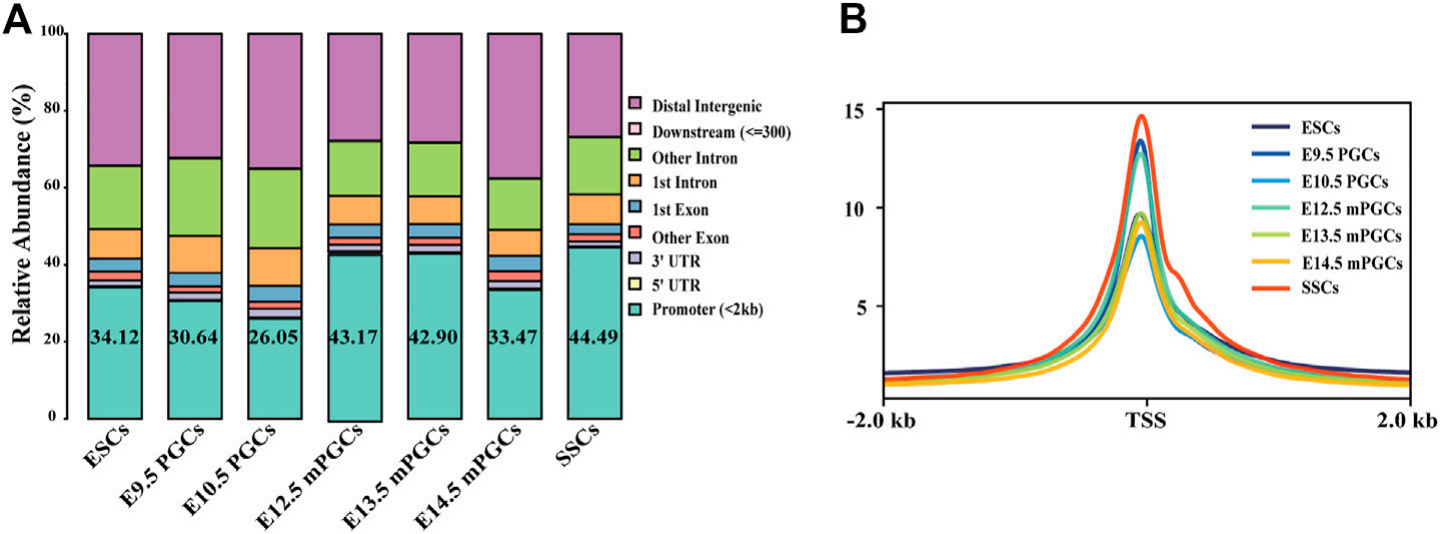
Identification of open chromatin regions. **(A)** Relative proportions of gene coding regions, introns, exons, and upstream and downstream regions in mouse ESCs, PGCs, and SSCs. **(B)** Enrichment of peaks in the TSS region.

ATAC-seq and DNase-seq have emerged as powerful methods to study accessible chromatin and GRNs (Han et al., 2017; Maher et al., 2018). Thus, we obtained a comprehensive landscape of the accessible chromatin in mouse ESCs, PGCs, and SSCs. We first mapped the distribution map of transposase hypersensitive sites (THSs) or peaks by either ATAC-seq or DNase-seq data. Figure 2A summarizes the relative enriched proportions of promoter, 5′ UTR, 3′ UTR, exons, downstream regions, and distal intergenic regions. Most of the peaks in ESCs, PGCs, and SSCs were found to be located within the promoter (peaks were located within 2 kb upstream of transcriptional start site (TSS)) and distal intergenic regions. The proportion of promoters in the SSCs was the highest (44.49%), while the proportion of promoters on embryonic day (E) 10.5 PGCs was the lowest (26.05%). We next examined the signal of peaks located within 2 kb of TSS using average plots (Figure 2B). The results revealed that a large proportion of peaks are located close to TSS, which means that the TSS tends to bind to TFs.

### Specific genes and specific TFs of mouse ESCs, PGCs, and SSCs

**FIGURE 3:**
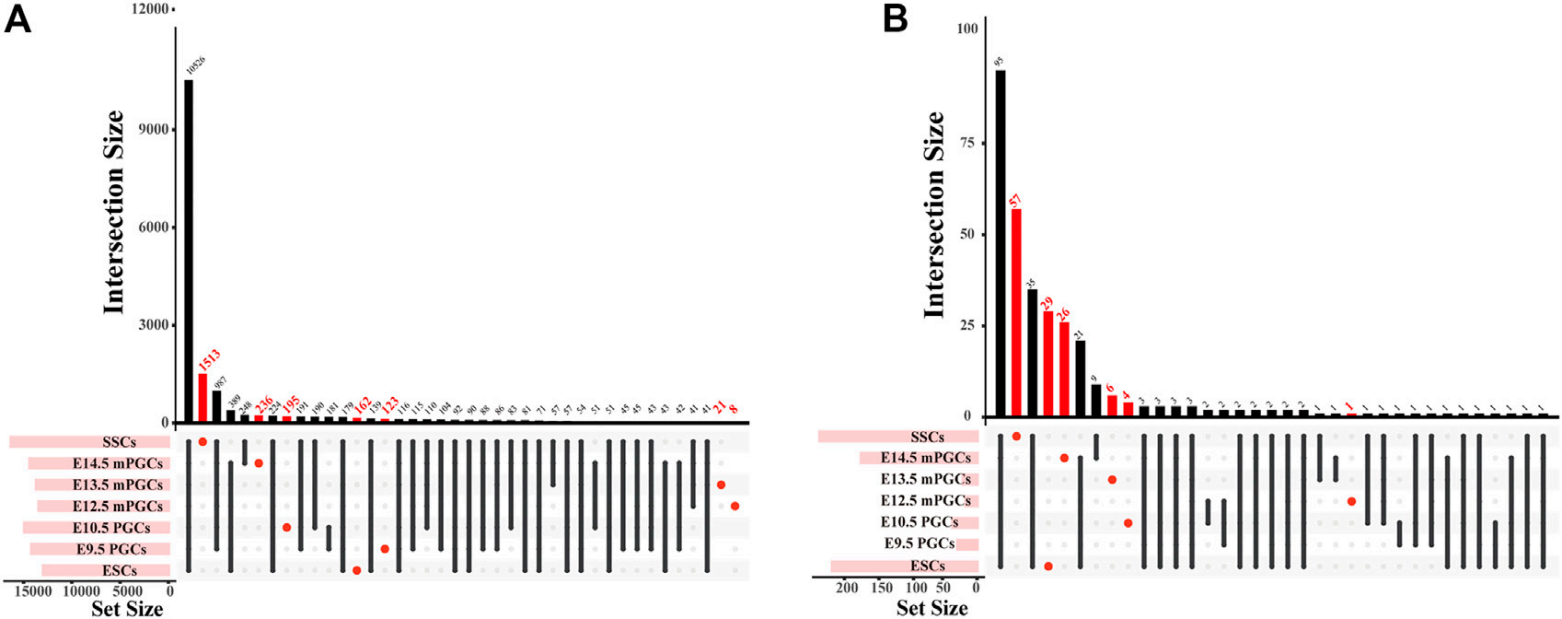
The number of cell-specific genes and cell-specific TFs in mouse ESCs, PGCs, and SSCs. **(A,B)** UpSet plot of cell-specific genes **(A)** and cell-specific TFs **(B)**.

The identified peaks can be used to predict motifs generally recognized by TFs, and GRNs of the cells can also be revealed through chromatin profiling assays (Nikolaev et al., 2007). Accordingly, we first obtained cell-specific genes and cell-specific TFs and then preliminarily constructed cell-specific GRNs. A total of 2,258 cell-specific genes were identified from DNase-seq and ATAC-seq for mouse ESCs, PGCs, and SSCs (Figure 3A, Supplementary Table S2). The largest number of cell-specific genes was expressed in SSCs, while the fewest cell-specific genes were expressed in E12.5 male (m) PGCs (Figure 3A). We also scanned the DNA-binding motifs and their associated TF motifs by using HOMER. We illustrate the total number of TF motifs that can be found across mouse ESCs, PGCs, and SSCs (Supplementary Table S2), and the distribution of TF motifs across the cell subsets is shown in Figure 3B. The ascending order ranked by the number of cell-specific TF motifs expressed was E9.5 PGCs, E12.5 mPGCs, E10.5 PGCs, E13.5 mPGCs, E14.5 mPGCs, ESCs, and SSCs. Of these TFs, no cell-specific TFs were found in E9.5 PGCs. The results above imply the different developmental stages of male germ cells as the level of analysis because genes and TFs can vary in spatial and temporal expression.

### TF-mediated gene regulatory networks of mouse ESCs, PGCs, and SSCs

**FIGURE 4:**
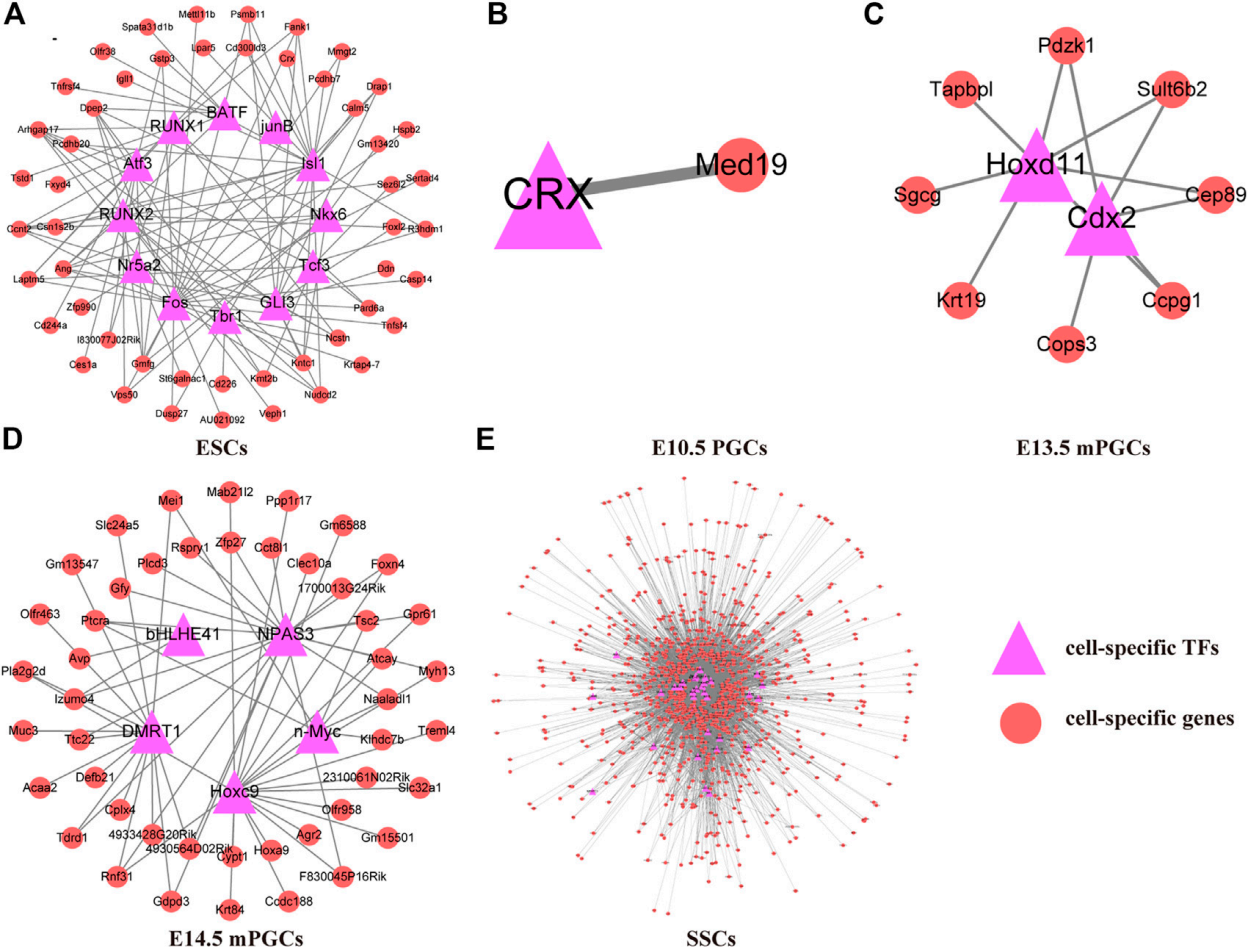
TF-mediated GRNs of ESCs, PGCs, and SSCs. **(A)** ESCs. **(B)** E10.5 PGCs. **(C)** E13.5 mPGCs. **(D)** E14.5 mPGCs. **(E)** SSCs.

To preliminarily construct the TF-mediated GRNs of mouse ESCs, PGCs, and SSCs, we analysed the cell-specific genes related to the cell-specific TF motifs according to ChIP-seq. We found that GRNs of mouse ESCs, PGCs, and SSCs differ between different cell types. Therefore, 50 cell-specific genes are targeted by cell-specific TFs in ESCs (Figure 4A), and only one cell-specific gene is targeted by cell-specific TFs in E10.5 PGCs (Figure 4B). E13.5 mPGCs (Figure 4C) and E14.5 mPGCs (Figure 4D), containing 8 and 46 cell-specific genes, respectively, were targeted by cell-specific TFs. However, no cell-specific genes are targeted by cell-specific TFs in E9.5 PGCs and E12.5 mPGCs. We also found that SSCs had the largest number of cell-specific TFs and their target genes, and 958 cell-specific genes were targeted by 33 cell-specific TFs, comprising 7,326 regulatory relationships in SSCs, after removing the redundant and unannotated TFs (Supplementary Table S3, Figure 4E). Taken together, these results showed that cell-specific TFs and their targeting cell-specific genes are different between mouse ESCs, PGCs, and SSCs. The results above implied that cell-specific TFs and their targeted cell-specific genes in mouse ESCs, PGCs, and SSCs could be used to determine their identity.

### WGCNA of the SSC-specific genes associated with SSC-Specific TFs

**FIGURE 5:**
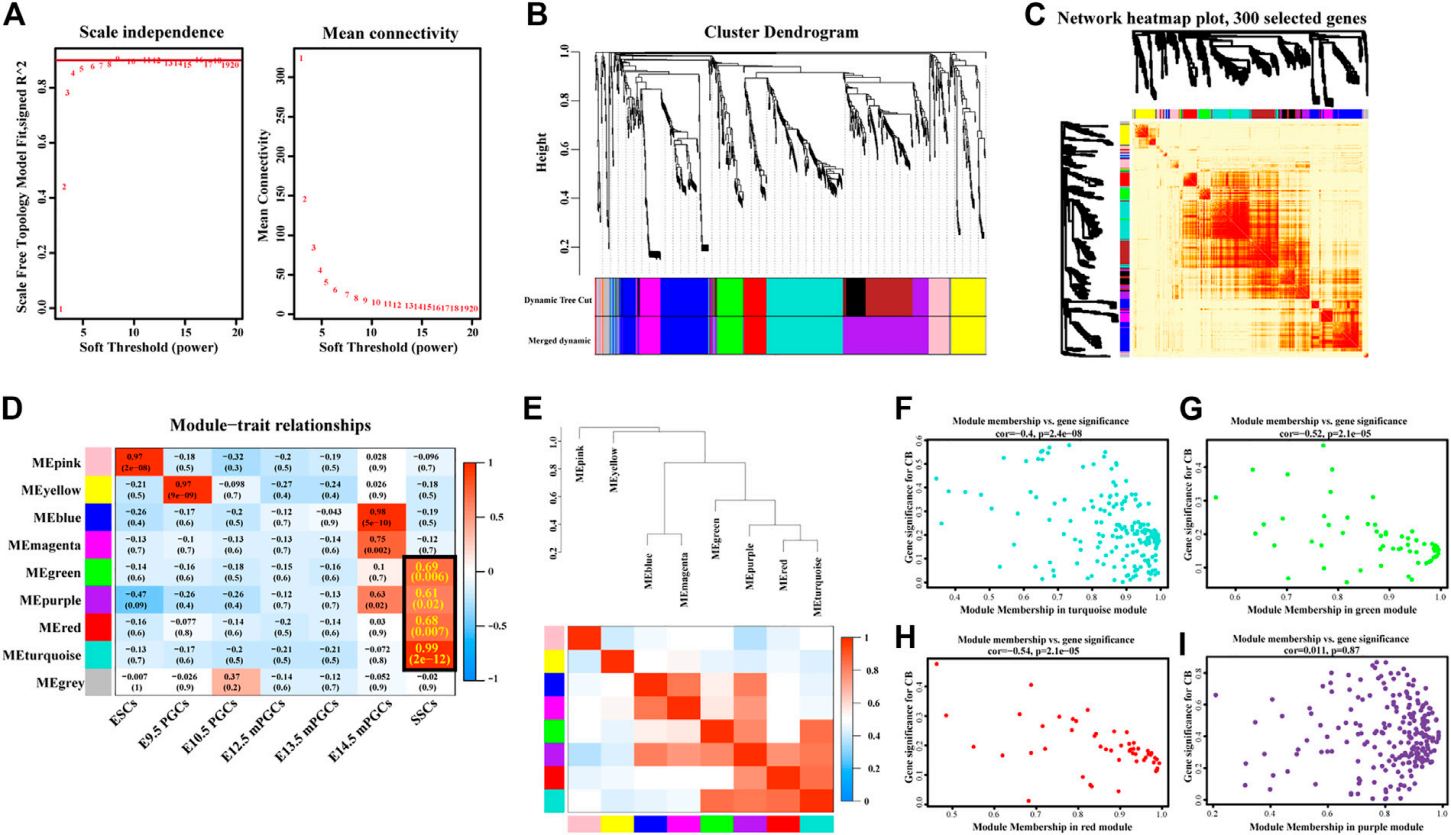
Construction of coexpression modules by WGCNA. **(A)** Scale-free index analysis for soft-threshold power and mean connectivity analysis for various soft-threshold powers. **(B)** Hierarchical clustering tree was developed by the weighted correlation coefficients. Each branch represents a coexpression module in different colours. **(C)** Interaction relationship analysis of coexpressed genes. The branch in the hierarchical clustering dendrograms corresponds to each module. Different colours of the horizontal axis and vertical axis represent different modules. The more saturated red indicates the higher coexpression interconnectedness in the heatmap. **(D)** Heatmap of the correlation between modules and hallmark gene sets. The framed turquoise, green, red, and purple modules were the most positively correlated with SSCs. Gene significance (GS) and its corresponding *p-value* were computed and are shown in the heatmap. **(E)** Hierarchical clustering of module hub genes that summarize the modules yielded in the clustering analysis (top) and heatmap plot of the adjacencies in the hub gene network (below). **(F–I)** Scatter plot of the GS for the grade vs. the MM in the turquoise **(F)**, green **(G)**, red **(H),** and purple **(I)** modules.

Previously, we learned that 958 SSC-specific genes are targeted by 33 SSC-specific TFs, comprising 7,326 regulatory relationships. To further explore the possible target genes for SSC-specific TFs and reveal the hub SSC-specific genes, we conducted WGCNA based on the RNA-seq (Supplementary Table S1) results. We first identified the relatively balanced scale independence and mean connectivity of the WGCNA, and the soft threshold β = 9 was adopted to achieve the scale-free topology criterion of the network (Figure 5A) and then obtained a hierarchical clustering tree using the dynamic cutting method (Figure 5B). The 9 modules marked were identified, and the interactions of the 9 modules were visualized as a network heatmap. The results revealed that each module was an independent validation to each other, which indicated that genes in the same module had a highly coexpressed relationship with each other (Figure 5C). The turquoise, green, red, and purple modules were significantly positively (*p-value* < 0.05) correlated with the SSCs, indicating that the turquoise, green, red, and purple modules may play an important role in the formation of SSCs (Figure 5D). Of these, the turquoise module was the most positively correlated with SSCs, including 181 genes, the green module contained 60 genes, the red module contained 55 genes and the purple module contained 127 genes. However, no module was significantly negatively correlated with the SSCs (Figure 5D). Then, we calculated eigengenes and clustered them according to their correlation to explore the coexpression similarity of all modules, and similar results were demonstrated by the heatmap plotted according to adjacencies (Figure 5E). Next, we evaluated the correlation between the gene significance (GS) and module membership (MM) in the turquoise, green, red, and purple modules. The correlation was significant in the turquoise (*R* = −0.4, *p-value* = 2.4e-08), green (*R* = −0.52, *p* = 2.1e-05), and red (*R* = −0.54, *p-value* = 2.1e-05) modules (Figures 5F–H), but no significant in the purple (*R* = 0.011, *p-value *= 0.87) module (Figure 5I).

### Hub genes, GO enrichment and KEGG pathway analyses of the turquoise and red modules

**FIGURE 6:**
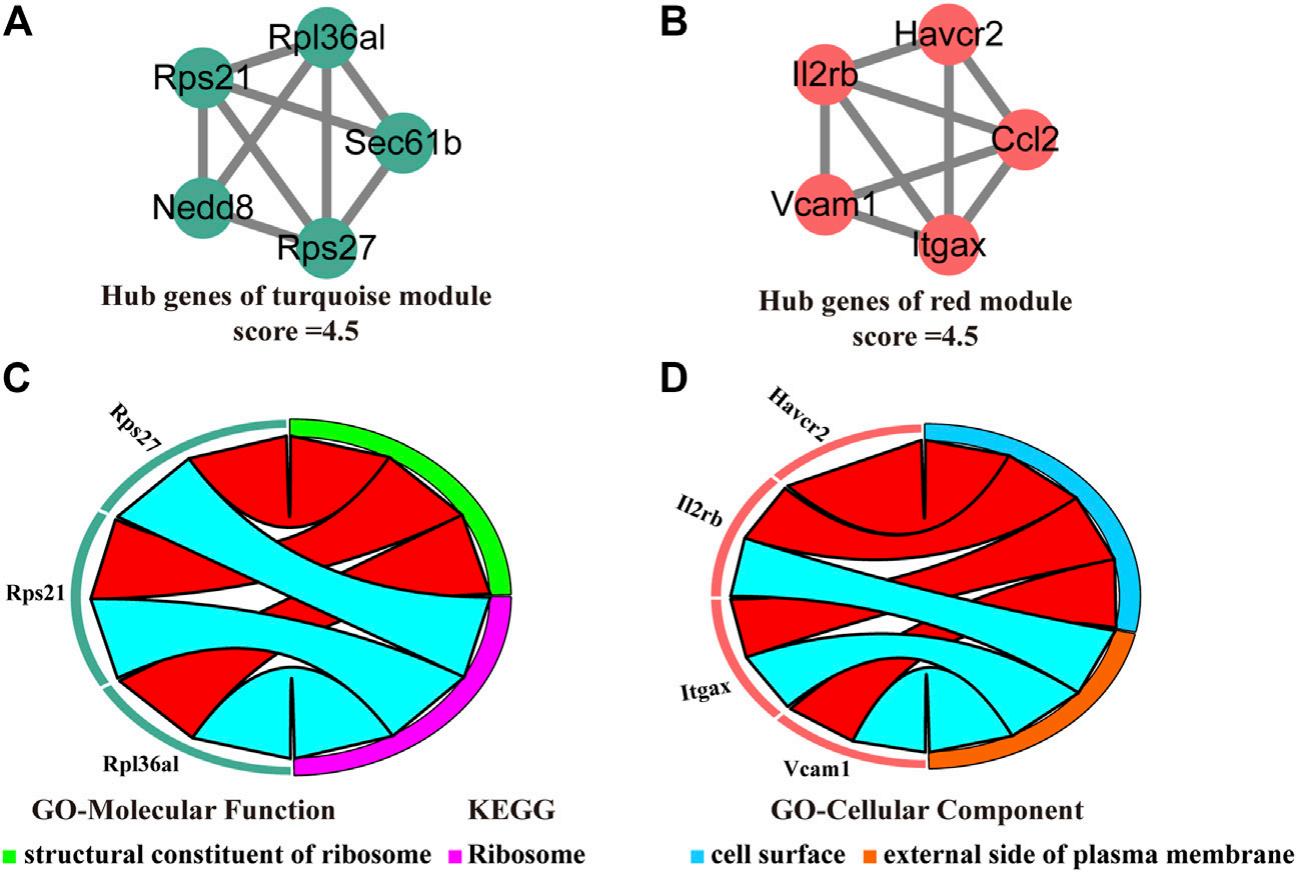
Identification, enrichment, and interrelation analysis of the hub SSC-specific genes. **(A,B)** The hub SSC-specific genes in the turquoise **(A)** and red modules **(B)**. **(C,D)** GO enrichment and KEGG pathway analysis of the hub genes in the turquoise **(C)** and red modules **(D)**.

We visualized the turquoise (Figure 6A) and red (Figure 6B) as networks in MCODE and screened out significant modules (score ≥ 4.5). However, the green and purple module are not a significant module in MCODE. Subsequently, the functional annotation for the hub genes in the turquoise and red modules was performed using DAVID. As the results show, the hub genes of the turquoise module were primarily enriched in structural constituent of ribosome (GO-Molecular Function) and ribosome (KEGG, Figure 6C). The hub genes of the red module, on the other hand, the results of enrichment analysis were mainly concerned with the cell surface and external side of plasma membrane (GO-Cellular Component, Figure 6D).

## Inferring key SSC-Specific TFs and constructing key SSC-Specific TF-Mediated GRNs

**FIGURE 7:**
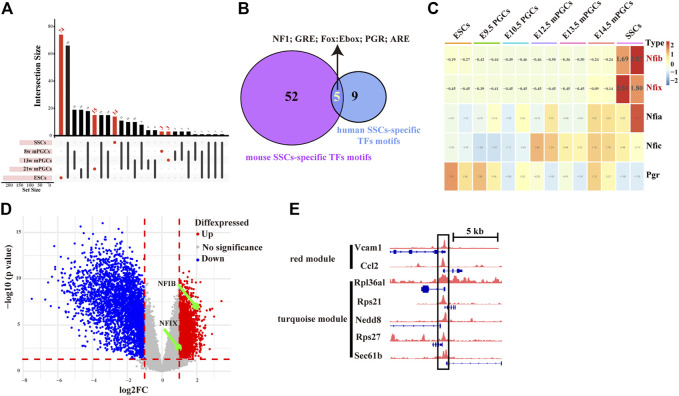
Screening of key SSC-specific TFs and their targeting hub SSC-specific genes. **(A)** UpSet diagram showing the number of cell-specific TF motifs in human ESCs, PGCs, and SSCs. **(B)** Venn diagram showing overlapping SSC-specific TFs between humans and mice. **(C)** Heatmap showing changes in the gene expression of overlapping SSC-specific TFs. The red colour in the heatmap indicates high expression, and the green colour indicates low expression. **(D)** Volcano plot of differentially expressed genes between testis samples with impaired spermatogenesis and testis samples with normal spermatogenesis. **(E)** IGV screenshots of ChIP-seq data for NFIB at the hub SSC-specific genes of the turquoise module (*Rpl36al*, *Rps27*, *Rps21*, *Nedd8*, and *Sec61b*) and the red module (*Vcam1* and *Ccl2*).

To assess whether SSC-specific TFs in mice also have roles in human SSCs, we further screened key SSC-specific TFs. Combining the landscape of the accessible chromatin in human ESCs, PGCs, and SSCs (Figure 7A), we finally obtained five overlapping SSC-specific TF motifs, including NF1 family TF motifs (NFIA, NFIB, NFIC, and NFIX), GRE, Fox:Ebox, PGR, and ARE, whereas no overlapping ESC-specific TF motifs and PGC-specific TF motifs were found (Figure 7B). Of these TFs, *Nfib* and *Nfix* exhibited abnormally high gene expression levels relative to mouse ESCs and PGCs (Figure 7C). Moreover, we also found, from the analysis of public databases (GSE145467), that *Nfib* (log2-fold change (*log2FC*) = 2.19, *p-value* = 3.38E-07) and *Nfix* (*log2FC* = 1.24, *p-value* = 1.15E-03) were upregulated in testis samples with impaired spermatogenesis in comparison with testis samples with normal spermatogenesis (Figure 7D). Finally, to construct the key SSC-specific TF-mediated GRNs, we found in the ChIP-seq database (Cistrome Data Browser: 69,127) that NFIB most likely targeted the hub SSC-specific genes of the turquoise module (*Rpl36al*, *Rps27*, *Rps21*, *Nedd8*, and *Sec61b*) and the red module (*Vcam1* and *Ccl2*). (Figure 7E). However, for NFIX, no ChIP-Seq data were available. The results preliminarily implied that key SSC-specific TFs could be involved in the regulation of SSC formation and spermatogenesis by regulating their mediated GRNs. Nevertheless, experimental validation is required for confirmation.

## Discussion

Currently, it is difficult to elucidate the mechanisms of human SSC formation because of the long period of the development process *in vivo* and restricted ethical issues (Martin and Seandel, 2013; Sohni et al., 2019). Alternatively, studies on the differentiation of ESCs to SSCs *in vitro* might be useful to understand human SSC formation and male infertility (Martin and Seandel, 2013; Li et al., 2019). Over the past years, a wide range of mouse models and *in vitro* cell culture systems have been used to explore the formation mechanisms of SSCs, inducing ESCs into PGCs and SSCs through transgenic technologies or by adding cytokines and chemical induction reagents to the culture medium (Geijsen et al., 2004; Wan et al., 2014; Li et al., 2019). However, the low efficiency and undefined induction factors of *in vitro* induction have always been important factors restricting this technology (Nagamatsu and Hayashi, 2017). Therefore, it is urgent to find key factors that influence the differentiation of ESCs into SSCs *in vitro* from a new perspective. In this study, by combining chromatin property data (ATAC-seq, DNase-seq, ChIP-seq) and gene expression data (RNA-seq, microarray data), cell-specific TFs and cell-specific TF-mediated GRNs in the process of SSC formation were identified. As a result, several key SSC-specific TFs and their targeting hub SSC-specific genes were found to potentially be involved in regulating the differentiation of ESCs into SSCs *in vitro*.

By analysing the accessible chromatin of mouse ESCs, PGCs, and SSCs, we found that a large proportion of peaks were located close to TSS, and the spatial and temporal expression of genes varied in different developmental stages of male germ cells. Moreover, the integration of chromatin property data and gene expression data can contribute to decrypting transcriptional regulatory codes of male germ cell formation. In our analysis, through integrated chromatin property data (ATAC-seq, DNase-seq, ChIP-seq) and gene expression data (RNA-seq, microarray data), we found overlapping SSC-specific TFs between humans and mice and constructed TF-mediated GRNs in the process of SSC formation. It has previously been shown that the roles of TFs in GRNs are almost identical between humans and mice (Stergachis et al., 2014; Yue et al., 2014). Interestingly, Kim et al. found that the rewiring of GRNs contributes to the phenotypic discrepancies that occur between humans and mice (Ha et al., 2022).

We obtained five overlapping SSC-specific TF motifs between humans and mice, including NF1 family TF motifs (NFIA, NFIB, NFIC, and NFIX), GRE, Fox:Ebox, PGR, and ARE. Therein, *Nfib* and *Nfix* exhibited abnormally high gene expression levels relative to mouse ESCs and PGCs. Furthermore, *Nfib* and *Nfix* were upregulated in the testis sample with impaired spermatogenesis in comparison with the testis sample with normal spermatogenesis. These results suggest that NFIB and NFIX could be involved in the regulation of ESCs differentiation into SSCs and spermatogenesis.

The nuclear factor one (NFI) family of DNA binding proteins, previously also known as CCAAT box-binding transcription factors or TGGCA-binding proteins, has four members in vertebrates: NFIA, NFIB, NFIC, and NFIX (Borgmeyer et al., 1984; Zenker et al., 2019). In mice, *Nfib* and *Nfix* expression is most pronounced within the dorsal telencephalon and cerebellum. *Nfib* and *Nfix* knockout mice display severe brain phenotypes, suggesting that *Nfib* and *Nfix* are critical for brain development (Campbell et al., 2008; Bunt et al., 2015; Zenker et al., 2019). Other studies have found that *Nfib* knockout mice exhibit lung defects (Steele-Perkins et al., 2005), and *Nfic* knockout mice have abnormal teeth (Steele-Perkins et al., 2003). In humans, NFIB and NFIX have been related to human development and cancer (Zenker et al., 2019). NFIB and NFIX have been shown to act as either oncogenes or tumour suppressors across various cancers (Denny et al., 2016; Fane et al., 2017; Rahman et al., 2017; Zhao et al., 2021). Interestingly, evidence shows that NFIB is a key epigenetic regulator during development and within lung cancer (Denny et al., 2016). This study found that NFIB promotes metastasis of human small cell lung cancer (SCLC) cells through a widespread increase in chromatin accessibility (Denny et al., 2016). It has been reported that NFIX interference in human SSCs stimulates propagation and suppresses early apoptosis of human SSCs; additionally, a study also found that NFIX negatively controls cyclin A2, cyclin B1, and cyclin E1 in human SSCs (Zhou et al., 2018). During PGCs development, it has been reported (Li et al., 2018) that the chromatin of mitotic-arrested male PGCs is permissive through nuclear transcription factor Y (NFY) binding in the distal regulatory regions, in contrast to that of meiotic female PGCs.

Notably, by analysing NFIB ChIP-seq data from the Cistrome database, we found that NFIB targets hub SSC-specific genes of the turquoise module (*Rpl36al*, *Rps27*, *Rps21*, *Nedd8*, and *Sec61b*) and the red module (*Vcam1* and *Ccl2*). *Rpl36al* is one of the X-linked human genes encoding ribosomal proteins. *Rpl36al* lacks introns in its coding regions, which was likely retrotransposed from X-linked genes (Uechi et al., 2002). Studies have found that *Rpl36al* may serve as a diagnostic biomarker based on immune infiltrates in Alzheimer’s disease (Liu et al., 2021). Ribosomal protein S27 (RPS27) and RPS21 are part of the ribosomal protein. RPS27 might play a role in the initiation of translation (Feldheim et al., 2020). Furthermore, RPS27 protein was specifically expressed in tumour cells and neurons but not in healthy astrocytes (Feldheim et al., 2020). In contrast, fewer studies have examined RPS21, and a recent study found that RPS21 plays an essential role in the invasive behaviour of osteosarcoma cells through the inactivation of the mitogen-activated protein kinase (MAPK) pathway (Wang et al., 2020). Neural precursor cell expressed developmentally downregulated-8 (NEDD8) is a ubiquitin-like molecule that can be transferred to substrates to regulate protein function through a process termed protein neddylation (Kamitani et al., 1997), and *Nedd8* is expected to play a role as a therapeutic target in cancer (Jiang et al., 2020). The Sec61 translocon subunit beta (SEC61B) complex is the central component of the protein translocation apparatus of the endoplasmic reticulum membrane (Feng et al., 2017). Studies have reported that *Sec61b* was newly detected as a candidate gene involved in ovarian clear cell carcinogenesis (Yamada et al., 2021). Unlike the above genes, in addition to being involved in a range of cancers and diseases, *Vcam1* and *Ccl2* might play a crucial part in testicular interstitial tissues. Recently, among the 3D-reaggregated cultures of dissociated testicular cells from prepubertal mice, it was found that VCAM1, one of the ligands for integrins α4β1 and α9β1, is expressed mainly in CD34^+^ cells and adult Leydig cells but not in foetal Leydig cells, implying that the VCAM1-α4β1 integrin interaction plays an important role in the formation of testicular interstitial tissues *in vitro* and *in vivo* (Abe et al., 2021). In autoimmune orchitis, as a chemotactic factor, CCL2 can induce attraction and extravasation of immune cells within the testicular interstitium (Guazzone et al., 2009). Although no studies have reported that these hub SSC-specific genes are involved in the differentiation of ESCs into SSCs, our results may provide some evidence for this aspect.

There are limitations in the study. First, we obtained five overlapping SSC-specific TF motifs between humans and mice, whereas no overlapping ESC-specific TF motifs and PGC-specific TF motifs were found. Thus, subsequent studies focused on the SSC-specific genes and associated SSC-specific TFs. Furthermore, we found that the overlapping SSC-specific TFs (NFIB and NFIX) exhibited abnormally high gene expression levels relative to ESCs and PGCs, but the expression level of *Nfib* and *Nfix* did not show an obvious change between ESCs and PGCs. These results may reflect the regulatory network from PGCs to SSCs, but not the upstream regulation from ESCs to PGCs. Second, no NFIB and NFIC ChIP-seq data from the mouse SSCs were found by searching the NCBI-GEO and PubMed. Ultimately, we found in a ChIP-seq database (Cistrome Data Browser: 69,127) that NFIB most likely targeted the hub SSC-specific genes of the turquoise module (*Rpl36al*, *Rps27*, *Rps21*, *Nedd8*, and *Sec61b*) and the red module (*Vcam1* and *Ccl2*). It is undeniable that the evidence was not sufficient enough to use the ChIP-seq database (Cistrome Data Browser: 69,127) comes from the mammary gland. These results may only provide suggestions for future research.

## Conclusion

In conclusion, after a comprehensive analysis of chromatin property data (ATAC-seq, DNase-seq, ChIP-seq) and gene expression data (RNA-seq, microarray data), we preliminarily identified SSC-specific TFs and constructed TF-mediated GRNs in the process of SSC formation. The key SSC-specific TFs (NFIB, NFIX) and their targeting hub SSC-specific genes were specifically analysed. The results also imply that NFIB and NFIX could be involved in the regulation of SSC formation and spermatogenesis. Our investigation establishes a foundation for future research aiming to elucidate the role of these TFs and their targeting hub genes in SSC formation and provides potential induction factors for further optimizing the induction efficiency of the differentiation of ESCs into SSCs *in vitro*.

## Data Availability

The original contributions presented in the study are included in the article/Supplementary Material, further inquiries can be directed to the corresponding author.
