# Supplementary material for: Integrated bioinformatics analysis of the transcription factor-mediated gene regulatory networks in the formation of spermatogonial stem cells
Source: Front Physiol. 2022 Dec 8;13:949486. doi: 10.3389/fphys.2022.949486 (PMC9773208; doi:10.3389/fphys.2022.949486)
Supplement: Supplementary file 2 [file Table1.DOCX]

Supplementary Material

**Supplementary Table 1.** The detailed information for each dataset.

| **GEO accession** | **Samples** | **Type** |
| --- | --- | --- |
| GSE178551 | 2 mouse ESC | ATAC-seq |
| GSE109767 | 2 mouse E9.5 PGCs;  2 mouse E10.5 PGCs;  2 mouse E12.5 mPGCs^1^;  2 mouse E13.5 mPGCs;  2 mouse E14.5 mPGCs | DNase-seq |
| GSE102954 | 2 mouse SSCs | ATAC-seq |
| GSE120648 | 2 human ESCs | ATAC-seq |
| GSE109768 | 2 human 8w mPGCs^2^;  2 human 11w mPGCs;  2 human 13w mPGCs;  2 human 21w mPGCs; | DNase-seq |
| GSE120507 | 2 human SSCs | ATAC-seq |
| GSE151444 | 2 mouse ESCs | RNA-seq |
| GSE94136 | 2 mouse E9.5 PGCs;  2 mouse E10.5 PGCs;  2 mouse E12.5 mPGCs;  2 mouse E13.5 mPGCs;  2 mouse E14.5 mPGCs | RNA-seq |
| GSE164604 | 2 mouse SSCs | RNA-seq |
| GSE145467 | 10 testis sample with normal spermatogenesis;  10 testis sample with normal spermatogenesis | microarray data |

^1^ mPGCs: male PGCs. ^2^ 8w mPGCs: 8-week male PGCs. The same below.

**Supplementary Table 2.** The number of cell-specific genes, cell-specific TFs, and cell-specific genes targeted by cell-specific TFs.

| **Type** | **Cell** | **Number** | **Numbers of cell-specific genes targeted by cell-specific TFs** |
| --- | --- | --- | --- |
| mouse cell-specific genes  (Total 2258) | ESCs | 162 | - |
|  | E9.5 PGCs | 123 | - |
|  | E10.5 PGCs | 195 | - |
|  | E12.5 mPGCs | 8 | - |
|  | E13.5 mPGCs | 21 | - |
|  | E14.5 mPGCs | 236 | - |
|  | SSCs | 1513 | - |
| mouse cell-specific TF motifs  (Total 123) | ESCs | 29 | 50 |
|  | E9.5 PGCs | 0 | 0 |
|  | E10.5 PGCs | 4 | 1 |
|  | E12.5 mPGCs | 1 | 0 |
|  | E13.5 mPGCs | 6 | 8 |
|  | E14.5 mPGCs | 26 | 46 |
|  | SSCs | 57 | 958 |
| human cell-specific TF motifs  (Total 109) | ESCs | 74 | - |
|  | 8w mPGCs | 15 | - |
|  | 13w mPGCs | 3 | - |
|  | 21w mPGCs | 3 | - |
|  | SSCs | 14 | - |
| mouse SSC-specific TF motifs ∩  human SSC-specific TF motifs | SSCs | 5 | - |
